# Series-Fed Microstrip Patch Antenna Array with Additive-Manufactured Foldable Honeycomb-Shaped Substrate †

**DOI:** 10.3390/mi15121449

**Published:** 2024-11-29

**Authors:** Sima Noghanian, Yi-Hsiang Chang, Patricio Guerron, Reena Dahle

**Affiliations:** 1 CommScope Ruckus Networks, Sunnyvale, CA 94089, USA; 2 Department of Technology, Illinois State University, Normal, IL 61761, USA; ychan13@ilstu.edu; 3 IBM, Poughkeepsie, NY 12601, USA; patricio.guerron@ibm.com; 4 Metamagnetics Inc., Marlborough, MA 01581, USA; rdahle@mtmgx.com

**Keywords:** foldable antenna, 3D-printed antenna, microstrip antenna, hinge

## Abstract

This paper presents a novel foldable S-band microstrip patch antenna array operating in the 2.4–2.45 GHz band. The substrate is designed to allow the array to be folded and arranged in tiles, forming a versatile, reconfigurable antenna array. Additive manufacturing is used to fabricate the substrate for ease of fabrication and flexibility in its design. The major challenge in this type of design is creating a proper method of feeding the elements while maintaining the array’s optimal performance. A novel hinge design that can hold a coaxial cable for the series-fed array is introduced. The hinge provides the capability of folding the array from a flat orientation into various folded orientations. In this paper, a 2 × 1 microstrip array unit is presented as proof of concept. The antenna was fabricated and measured, and the results of the measurements are in close agreement with the simulations. The antenna can provide a gain as high as 7.72 dBi in flat conditions.

## 1. Introduction

Foldable and flexible antennas have become essential components in portable and wearable electronics, as well as in applications requiring compact and adaptable wireless systems. Various applications of foldable antennas exist. For example, they could be used as a part of wearable wireless devices or integrated into drones or unmanned vehicle platforms, where antenna size and reconfigurability are critical design considerations. They may be used in small satellites since they can be folded during launch and deployed once in orbit. The interest in using a foldable antenna is usually due to limitations of size, weight, and power (SWaP). Foldable antennas can also offer multiple functionalities and can be reconfigured to have a different resonance frequency or radiation pattern. Traditional methods of accomplishing multifunctional reconfigurable antennas have included the use of varactors, MEMS, pin diodes, microfluidics, and liquid metals [1,2].

While there could be many innovative ways of making a foldable antenna, we focus on planar antennas implemented on a foldable structure in this paper. For these antennas, we would like to highlight three methods introduced in the literature, which include the following:

(1) Thick foldable materials, such as PCB-based antennas connected with conductive tape or corrugated hinges. One of the earliest demonstrations of this technique was given in [3], where a series-fed microstrip antenna was designed. Microstrip patches of different sizes were connected via curved microstrip lines. Thick Rohacell material was used behind the substrate, and the connection between the pieces of the ground plane was realized using thick conductive tape. A similar approach was discussed in [4]. A circularly polarized array of circular slotted patch antennas was proposed and fabricated on pieces of solid PCB materials. The array was fed through a corporate feed network, where it had to pass the connecting parts between the solid pieces. The connection was realized via metal tape. The folding was performed manually. A very similar approach was also proposed in [5]. In all the aforementioned literature, the authors used hybrid methods for the transition and hinges. In contrast, in this work, the authors propose an integrated bifunctional hinge that allows bending and connecting the microstrip lines.

(2) Mechanically actuated structures with movable components using push–pull or heat-sensitive hinges. In this category, the substrate of the microstrip antenna could be printed using additive manufacturing structures with moveable parts. For example, a combination of additive manufacturing and inkjet printing was reported in [6]. A planar structure was printed using 3D printing. The hinge was made of a shape-memory material that enabled folding the flat 3D-printed substrate into a cube shape. Microstrip patch antennas were made using inkjet printing conductive ink and individually fed through a coaxial probe. The inkjet printing method was also reported in [7] to print a series-fed microstrip array of three elements on paper that was then placed on top of a 3D-printed structure that could extend to support all three elements or was pushed to convert the structure to a single-element antenna. The connection between the elements was through microstrip lines.

(3) Origami-inspired configurations, where the antenna is mounted on foldable substrates made of flexible textiles or rigid materials like laminated printed circuit board (PCB) sections and 3D-printed components. Several designs are discussed in [8], which provides a summary and guidelines for designing origami antennas. In particular, a helical antenna designed using textile material is presented. The antenna is fed via connectors. A different way of using origami or foldable structures is to support the substrate with these structures that can be easily folded to improve the durability of the planar antennas. For example, 3D printing was used to create flexible substrates by combining ABS and NinjaFlex materials [9]. A high-permittivity material was used to 3D print an origami-shaped layer to be used under an air-substrate microstrip patch [10]. Its frequency and pattern were controlled by changing the substrate from fold to unfold states. A method to enhance the flexibility of the substrate was introduced in [11]. A load-bearing structure in the shape of an air-filled honeycomb periodic core layer was inserted between the antenna and the ground plane.

Each of these methods provides unique advantages for specific use cases but presents challenges in maintaining signal consistency, durability, and efficiency under folding or bending stress. Table 1 summarizes the approaches mentioned above.

**Figure 1:**
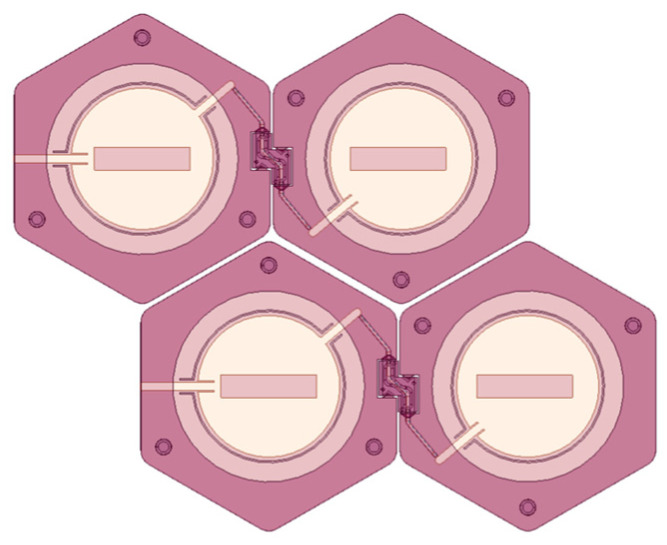
A honeycomb shape is suitable for creating 2D arrays.

This paper proposes a honeycomb-shaped antenna array using series-fed microstrip elements, designed to enhance flexibility and structural resilience. The honeycomb geometry offers versatile folding options from multiple directions and can be efficiently arranged into a 2D array (Figure 1). A primary challenge in such designs is the connection between antenna elements. The previous designs rely on individually fed elements or feedlines crossing over hinge points, which can reduce flexibility and resilience. A key innovation is embedding a coaxial cable within the hinge, which transitions seamlessly to a microstrip feed line, protecting the feed from physical stress and optimizing power transfer. This coaxial feed system ensures maximum flexibility, safeguarding the feed integrity during movements. By developing a hinge capable of housing the flexible coaxial cable and creating a seamless transition to a microstrip feed, our design achieves both the flexibility and durability necessary for advanced wearable and compact wireless applications.

## 2. Materials and Methods

### 2.1. Antenna Structure

The antenna was built using the 3D-printing method. PLA material was used as the substrate of the antenna. The PLA material properties were characterized to be a dielectric constant of $\varepsilon_{r}=$ 2.55 and a loss tangent of 0.01. The height of the substrate was assumed to be h = 60 mils (1.524 mm). Perhaps one of the simplest microstrip patch shapes suitable for a hexagonal substrate shape is a circular patch, whose radius may be calculated using Equations (1)–(4),

(1) $$W=\frac{c}{2f_{0}\sqrt{\frac{\varepsilon_{r}+1}{2}}}$$

(2) $$\varepsilon_{eff}=\frac{\varepsilon_{r}+1}{2}+\frac{\varepsilon_{r}-1}{2}\left[\frac{1}{\sqrt{1+12\frac{h}{W}}}\right]$$

(3) $$L=\frac{c}{2f_{0}\sqrt{\varepsilon_{eff}}}-0.824h\left(\frac{\left(\varepsilon_{eff}+0.3\right)\left(\frac{W}{h}+0.264\right)}{\left(\varepsilon_{eff}-0.258\right)\left(\frac{W}{h}-0.8\right)}\right)$$

(4) $$a=\frac{\sqrt{\left(W+h\right)\left(L+h\right)}}{2}$$

where $f_{0}$ is the resonance frequency and was set to 2.45 GHz. The patch radius a=21.5 mm. The width of the microstrip feedline was calculated to be 4.27 mm for a 50 Ω impedance. A hexagonal substrate with a side length of 44.64 mm was chosen to create the honeycomb shape.

**Figure 2:**
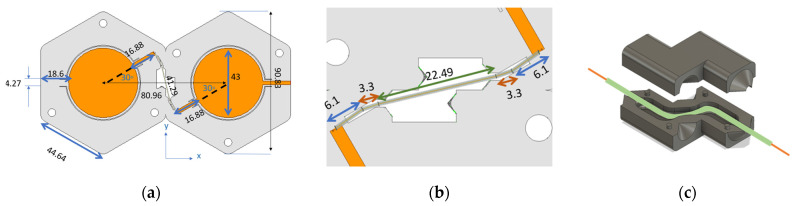
Circular patch two-element series-fed array designed for 2.45 GHz center frequency, (**a**) overall design, (**b**) coaxial cable connection, and (**c**) hinge holding the coax.

Based on this calculation, a two-element series-fed patch antenna array was fabricated (Figure 2). To connect the two patch elements, a combination of the microstrip line and coaxial cable was considered. The length of the combined microstrip line and coaxial cable was calculated to create a proper phase, resulting in in-phase antenna array elements. To model the coaxial cable, three cylindrical-shaped coaxial lines with a Teflon substrate were created with a radius of 0.34 mm for the outer cylinder and a radius of 0.12 mm for the inner one. The two end sections had a length of 6.1 mm, and the middle part consisted of two sections of 3.3 mm and one section of 22.49 mm, giving a total length of 41.29 mm. The center conductor was extended and united with the microstrip lines. The microstrip lines had a length of 16.88 mm. The combined length of the microstrip sections and the coaxial cable was 75.05 mm. This was approximately one wavelength (assuming a dielectric constant of 2.55, the wavelength was 76.68 mm). Figure 2a illustrates the fabricated antenna while on a flat surface, Figure 2b shows the cable model and length, and Figure 2c shows the hinge that houses the coaxial cable and protects it during bending.

### 2.2. Mechanical Design

**Figure 3:**
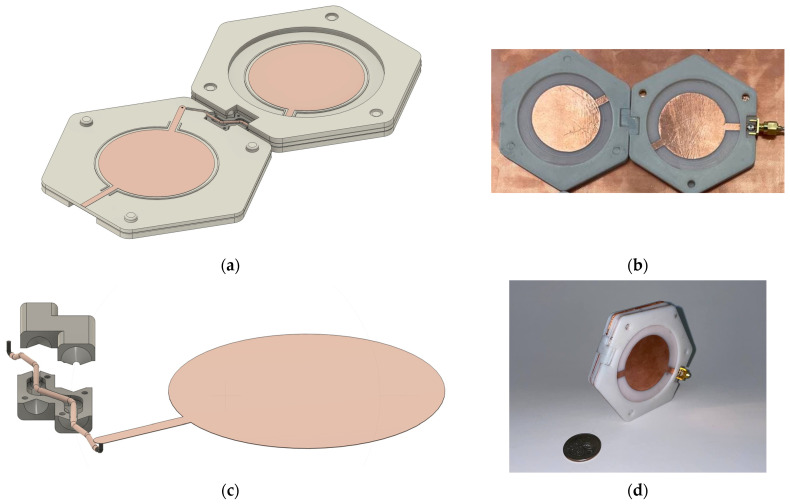
(**a**) Mechanical model of two-element antenna array, (**b**) fabricated antenna in flat orientation, (**c**) mechanical model of hinge and transmission line transition, and (**d**) fabricated antenna in 360° fold position.

Figure 3a illustrates the mechanical model of the two-element honeycomb antenna with 30° feedlines rendered in Autodesk Fusion 360 (version 2.0.19440). To ensure its performance and manufacturability, each antenna element was built with 3D-printed components, including a 3D-printed substrate carrying the antenna patch and ground, and sandwiched by two ‘ring-like’ hexagons for the structure rigidity needed by the hinge. The circular indents on the substrate helped align the antenna patches, while the three pins were used to guide the assembly, as shown in Figure 3b.

Figure 3c shows the hinge and transmission line connecting to an antenna patch. The top and bottom half of the hinge were printed separately to have a channel housing the coaxial cable. Two paired cone-shaped protrusions and cavities at each end of the hinge were used as the axes of rotation for the antenna cells. The transmission line model was created by sweeping three concentric cross-sections along the given path for the signal line, the insulation, and the ground. To properly model the transmission line, tangent conditions of different segments on the sweeping path were maintained with round corners to simulate the bending of the physical cable during the assembly process, and the three cross-sections for the transmission line needed to be perpendicular to the path. Figure 3d is a photo of the fabricated antenna in a 360° fold position.

**Figure 4:**
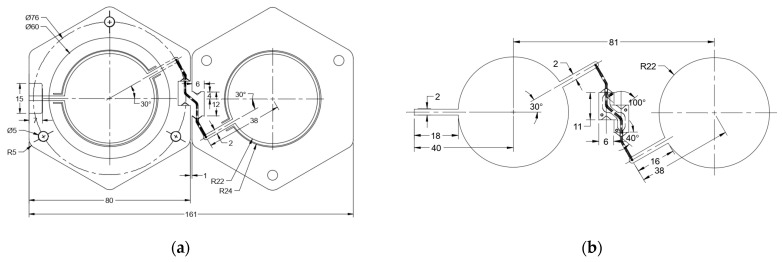
(**a**) Dimensions of honeycomb-shaped substrate and (**b**) dimensions of microstrip patch series-fed array and coaxial connection.

Figure 4a describes the critical dimensions of this two-element honeycomb antenna array. The dimensions of the honeycomb on the left are for the ‘ring-like’ structure, while those of the honeycomb on the right are for the patch antenna with a 30-degree feedline. The space between the two honeycomb substrates is where the hinge is located. Figure 4b shows the dimension of the hinge corresponding to two antenna patches with 30-degree feedlines.

### 2.3. Fabrication

Before its final assembly, the antenna’s components were fabricated with a fused deposition modeling (FDM) printer and a craft cutter, both of which were consumer-grade. The antenna substrate, ring structures, and hinge were fabricated with a Creality Ender-3 V2 3D printer (Creality 3D Technology Co, Ltd., Shenzhen, China). With a print volume of 220 × 220 × 250 mm^3^, this model uses a 1.75 mm nozzle with a print precision of ±0.1 mm. The diameter of the Comgrow brand PLA-based filament was 1.75 ± 0.03 mm. Once printed, the components were polished further to fit with each other. Table 2 represents the specific parameters used to print with the specific white-colored PLA filament.

The Silhouette Portrait 1 craft cutter (Silhouette America. Inc., Lindon, UT, USA) was used to cut the copper tape to form the antenna patches. This device allows an effective cutting area of 203 × 297 mm^2^, with a maximum media thickness of 31.5 mils or 0.8 mm, with a clearance of 2 mm. The maximum cutting force is 310 gf. Copper tape is not a usual material option for this specific model. The parameters used to configure this model were obtained by experimental practice to find the best cut without damaged edges. We found the parameters listed in Table 3 as the best configuration for cutting the copper tape.

## 3. Results

### 3.1. Original Antenna Simulation and Measurement Results

**Figure 5:**
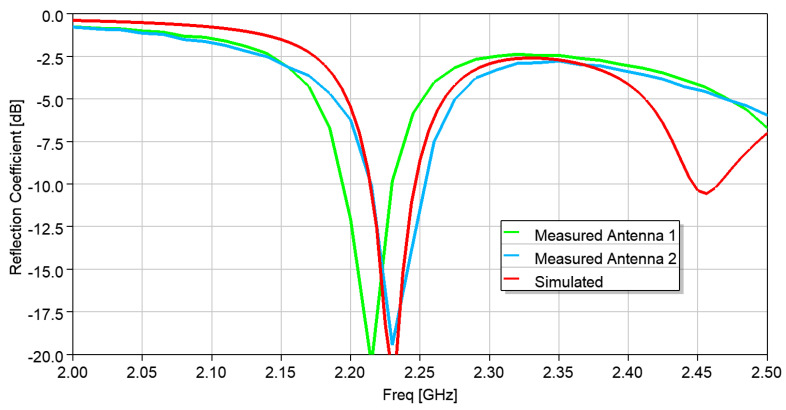
Comparison of the reflection coefficient of the series-fed 2-element patch array on a hexagonal substrate.

**Figure 6:**
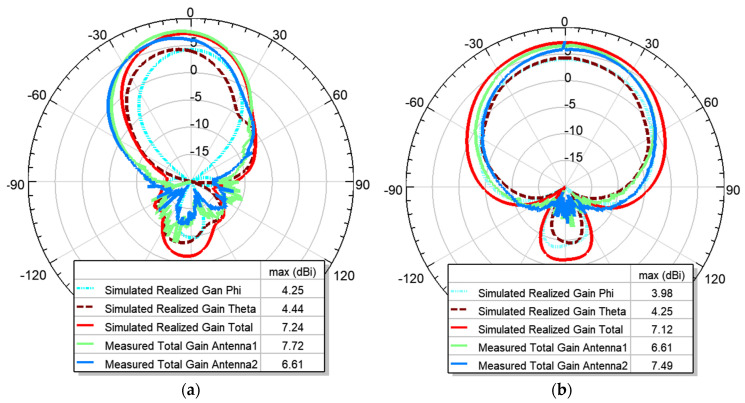
Simulated and measured radiated patterns at 2.24 GHz: (**a**) XOZ plane; (**b**) YOZ plane.

The antenna was modeled and simulated using the ANSYS HFSS 2022.R2 commercial electromagnetic simulation tool. Two samples of the antenna were then fabricated, “Antenna 1” and “Antenna 2”. The simulated and measured reflection coefficient (S_11_) are plotted in Figure 5 and are in close agreement, but the center frequency is at 2.24 GHz. As the equation for calculating the radius for a given frequency is approximate, and the effect of series feeding via the microstrip patch and coaxial line to the impedance was not included in these equations, this frequency shift is not unexpected. Nevertheless, the results are in excellent agreement. Considering the shift in the frequency, the radiation patterns were measured and compared with the simulated results at 2.24 GHz. Figure 6 shows the radiation pattern along the XOZ and YOZ planes, compared with the simulated ones, at 2.24 GHz.

**Figure 7:**
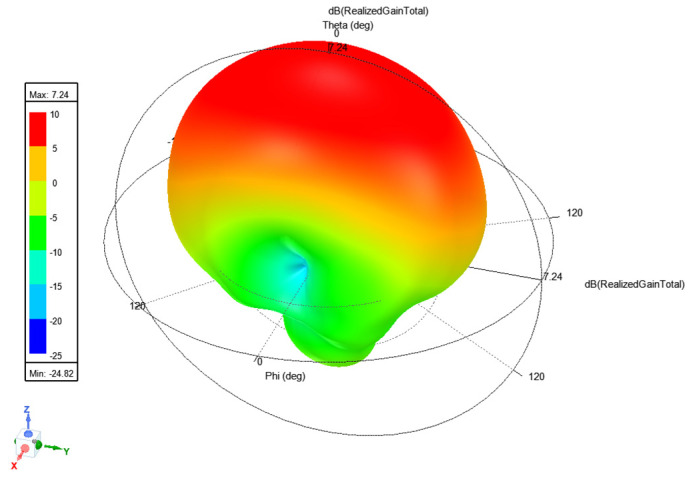
Simulated 3D realized gain at 2.24 GHz.

**Figure 8:**
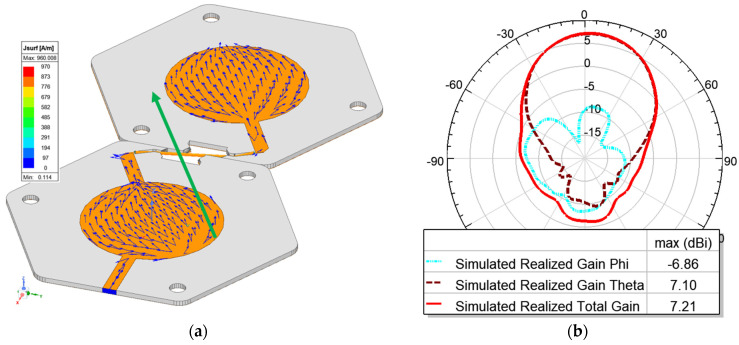
(**a**) Surface current distribution on the patches at 2.24 GHz; (**b**) radiation pattern φ and θ components on φ = 45°.

The 3D radiation pattern at 2.24 GHz is shown in Figure 7. The maximum realized gain in simulation is 7.24 dBi. The measured gain for Antenna 1 is 7.72 dBi, and for antenna 2, it is 6.61 dBi. A closer look at the φ and θ components of the gain on the XOZ and YOZ planes shows that although the total gain matches very well with the measured gain, the φ and θ components of the field are at a comparable level. This is in contrast with a single-element microstrip patch antenna. To investigate the reason for this tilted polarization, we looked at the current distribution of the patches, as shown in Figure 8. Figure 8a shows that that current is in the direction of the microstrip line connecting the two patches. This rotation of the microstrip lines causes the rotation in the polarization. However, the resulting field is still linearly polarized. To confirm this, the radiation patterns along φ = 45° are plotted in Figure 8b, and as expected, the φ component is significantly lower than the θ component.

### 3.2. Modified Antenna Simulation and Measurement Results

**Figure 9:**
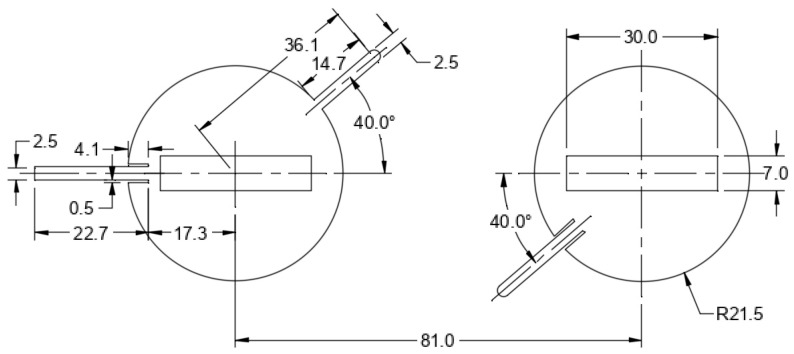
Inset-fed slotted 2-element honeycomb patch array slot dimensions.

**Figure 10:**
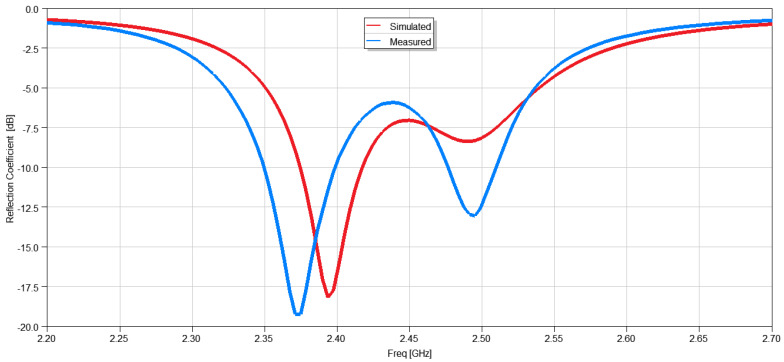
Measured vs. simulated reflection coefficients of the inset feed slotted patch antenna array.

To improve the polarization and rotate the E- and H-planes pattern along the XOZ and YOZ planes, respectively, a slot was introduced and added to the center of the patch. In addition, the feed line was modified to create an inset feed to improve the matching at 2.4 GHz. Figure 9 shows the modified design. The slot size is 7 mm × 30 mm. The feed line is also slightly rotated to create a 40° tilt with respect to the *X*-axis. The inset was optimized, and the final dimensions were 0.5 mm × 2.95 mm. Based on the proposed design, a 3D-printed two-element antenna was fabricated and measured. Figure 10 shows the simulated and measured reflection coefficients, and it is seen that the resonance frequency is 2.4 GHz. The bandwidth for the simulated S_11_ is from 2.38 to 2.42 GHz, and for the measured S_11_, it is from 2.35 to 2.40 GHz. The shift in the frequency could be due to the fabrication tolerances and estimation of the material properties of the substrate.

**Figure 11:**
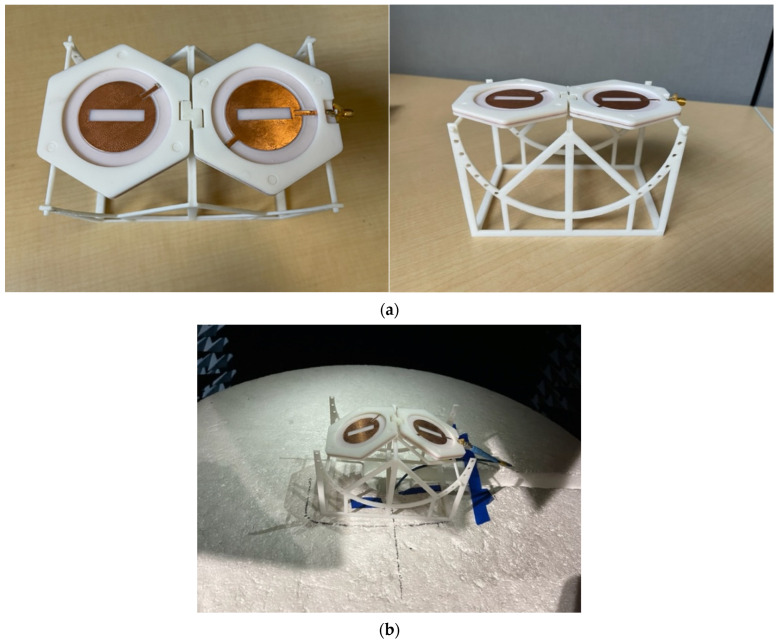
(**a**) Fabricated slotted patch antenna placed on a 3D-printed platform for measurements under flat and bent conditions; (**b**) flat and bent condition measurements in the anechoic chamber.

To fold the antenna at different angles, a 3D structure was designed to support folding the antenna toward the back side by increments of 10°. Figure 11a shows the 3D-printed antenna and the support structure. Figure 11b shows how the structure was used inside the anechoic chamber for the pattern measurements.

**Figure 12:**
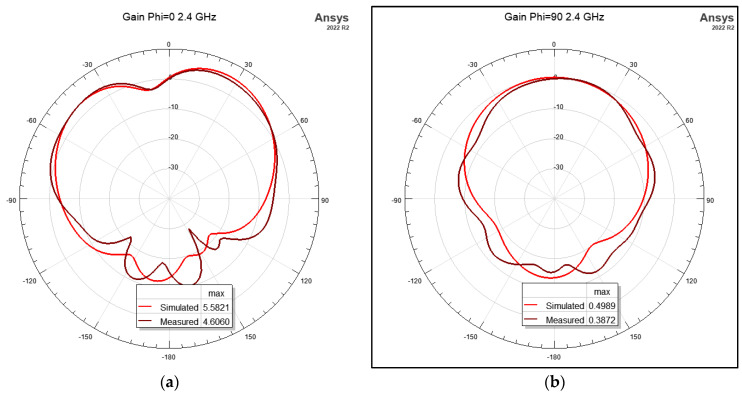
Simulated and measured radiation patterns of the inset-fed 2-element slotted patch at 2.4 GHz: (**a**) φ = 0°; (**b**) φ = 90°.

**Figure 13:**
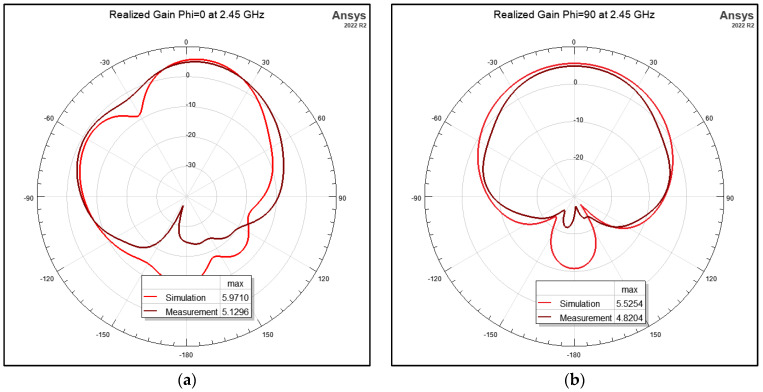
Simulated and measured radiation patterns of the inset-fed 2-element slotted patch at 2.45 GHz: (**a**) φ = 0°; (**b**) φ = 90°.

**Figure 14:**
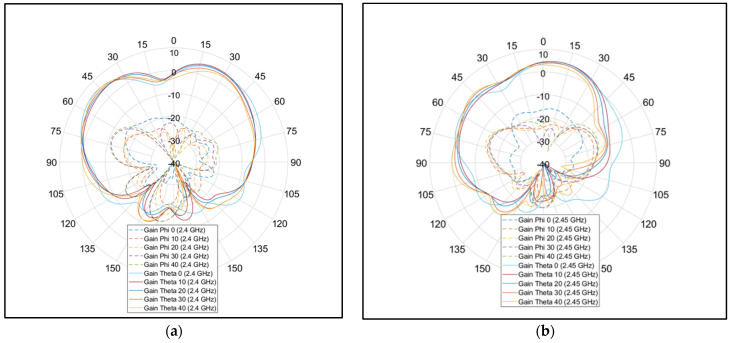
Measured radiation pattern at φ = 0° for various folding conditions. Dashed lines are the cross-polarization levels, and solid lines are the polarization patterns: (**a**) 2.4 GHz; (**b**) 2.45 GHz.

**Figure 15:**
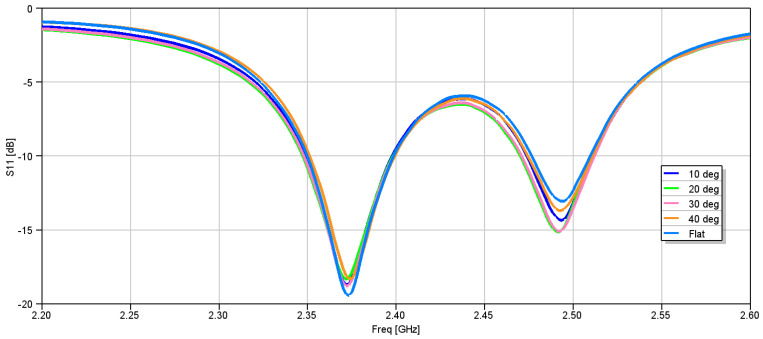
Measured reflection coefficients for various folding angles.

**Figure 16:**
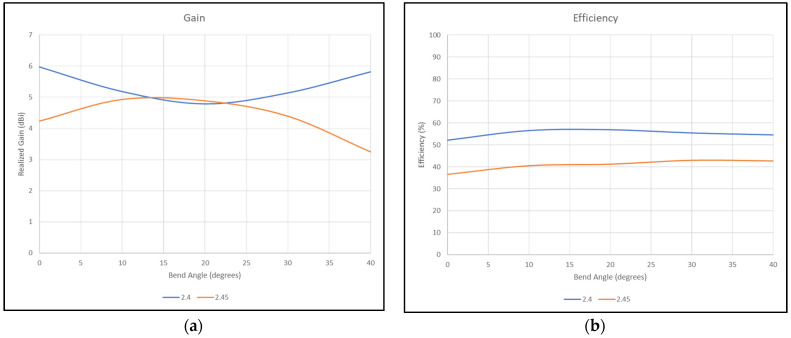
Measured (**a**) gain and (**b**) efficiency at 2.4 and 2.45 GHz vs. folding angle.

The flat antenna’s measured and simulated gain patterns are compared in Figure 12 at 2.4 GHz and Figure 13 at 2.45 GHz, respectively. The patterns agree well. In the next step, the antenna was folded back from 180° flat by increments of 10°. The co- and cross-polarization patterns were measured along the φ = 0° (XOZ) plane, where the bending was on that plane. The measurement results are shown in Figure 14 for the two frequencies of 2.4 GHz and 2.45 GHz. It is worth noting that the folding did not affect the input impedance significantly. Figure 15 shows the measured reflection coefficient for folding angles between 10° and 40°. Figure 16a shows the measured peak gain vs. the bending angle, and Figure 16b shows the measured radiation efficiency for the folding condition.

## 4. Discussion

**Figure 17:**
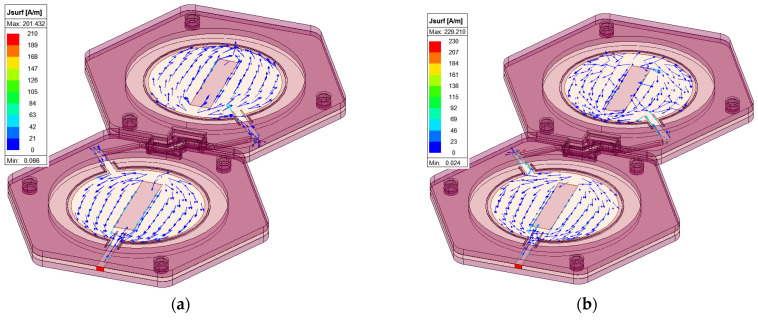
Surface current distribution of the slotted array at (**a**) 2.4 GHz and (**b**) 2.45 GHz.

Comparing the first and second designs, it is interesting to note that the original design provided a narrower beam. However, the main beam was rotated along the direction of the microstrip line that connected the first patch to the second patch. The narrower beam provided a higher gain. In the modified version with the added slot, the current was rotated to align with the slot, but this rotation also may create phase variations between the two slots. Figure 17 shows the current distribution for the slotted patch array. Comparing these current distributions with the one shown in Figure 8, the current distribution on each patch may not be in the same direction. This creates a wider beam with a notch in the broadside direction. Therefore, the slotted patch may be used in applications where a wider beam may be desirable. The side and shape of the slot can be used to modify and change the overall pattern.

## 5. Conclusions

This paper introduced a novel design of a foldable microstrip patch array with a honeycomb structure. One novel aspect of the design is to embed the coaxial cable in a hinge; therefore, the array can be completely folded without affecting the feedlines. The honeycomb shape is a good candidate for creating a two-dimensional array. While the simulation and measurements show excellent agreement, the design can be further studied by creating a two-dimensional array of two-sided elements, where one side is designed for one frequency and the other for a second frequency, to create a multifunctional foldable array.

## Figures and Tables

**Table 1:** Comparison of foldable or bendable microstrip antennas and array designs.

Ref.	Antenna Type	Fold Structure	Fabrication Method	Frequency (GHz)	Material	Feeding Method	Connection
[3]	Microstrip patch array	Manual fold	PCB	1.413	Rohacell	Curved microstrip lines	Conductive tape
[4]	Circular polarized microstrip array	Origami	PCB	3.0	PCB laminate	Microstrip line	Conductive tape
[5]	Microstrip array	Manual fold	PCB	2.5	PCB laminate	Microstrip lines over the hinge	Surrogate hinge
[6]	Microstrip	Heat-activated cube	Inkjet printing and 3D printing	2.4	VeroWhite plastic and silver nanoparticle ink	Coaxial cable	Shape-memory material (heat-activated)
[7]	Microstrip	Mechanical push	Inkjet printing and 3D printing	2.5	PLA, paper, and silver ink	Microstrip line	Microstrip line printed on paper
[8]	Helical	Origami	Cut and sew	0.4	Textile and conductive thread	Coaxial connector	NA
[9]	Microstrip single element	Heterogeneous substrate	3D printing and cutting	2.3	ABS and NinjaFlex, and copper tape	Coaxial connector	NA
[10]	Slotted microstrip	Mechanical	3D printing	Tunable 0.6–1.0	ABS and BaTiO_3_, and PCB	Slot-coupled microstrip line	NA
[11]	Microstrip array	Air-filled substrate	PCB	11.6–14.01	Polyamide and Nomex	Slot-coupled microstrip line	NA
This work	Microstrip array	Manual fold	3D printing and cutting	2.4	PLA and copper tape	Microstrip and coaxial cable	Coaxial embedded hinge

**Table 2:** Printer settings for Creality Ender-3 V2.

Parameter	Range	Configuration
Printing temperature	≤250 °C	210 °C
Build plate temperature	≤100 °C	60 °C
Infill density	10–100%	100%
Speed	≤180 mm/s	60 mm/s
Nozzle diameter	0.1 mm–0.4 mm	0.4 mm

**Table 3:** Settings for Silhouette Portrait Version 1.

Parameter	Range	Configuration
Angle of cut	1–10	6
Force	1–25	5
Speed	1–15	1
Passes	1–7	2
Type of cut	No cut, cut all line, cut edges	Cut edges

## Data Availability

The data generated in this research can be requested by contacting the corresponding author.

## References

[1] Noghanian S., Sharma S.K. (2021). Compact Reconfigurable Antennas. Multifunctional Antennas and Arrays for Wireless Communication Systems.

[2] Noghanian S., Sharma S.K. (2021). Radiation Pattern Reconfigurable Antennas. Multifunctional Antennas and Arrays for Wireless Communication Systems.

[3] Christodoulou C.G., Wahid P.F., Riad Mahbub M., Bailey M.C. (2000). Design of a Minimum-Loss Series-Fed Foldable Microstrip. IEEE Trans. Antennas Propag..

[4] Espinal F.A., Huff G.H., Pallampati S., Sessions D., Fuchi K., Bazzan G., Seiler S.R., Buskohl P.R., Cook A.B., Gillman A.S. (2020). Circularly-Polarised Origami-Inspired Folding Patch Antenna Sub-Array. IET Microw. Antennas Propag..

[5] Hamza M., Zekios C.L., Georgakopoulos S.V. (2020). A Thick Origami Reconfigurable and Packable Patch Array with Enhanced Beam Steering. IEEE Trans. Antennas Propag..

[6] Kimionis J., Isakov M., Koh B.S., Georgiadis A., Tentzeris M.M. (2015). 3D-Printed Origami Packaging with Inkjet-Printed Antennas for RF Harvesting Sensors. IEEE Trans. Microw. Theory Tech..

[7] Shah S.I.H., Lim S. (2017). Transformation from a Single Antenna to a Series Array Using Push/Pull Origami. Sensors.

[8] Georgakopoulos S.V., Zekios C.L., Sattar-Kaddour A., Hamza M., Biswas A., Clark B., Ynchausti C., Howell L.L., Magleby S.P., Lang R.J. (2021). Origami Antennas. IEEE Open J. Antennas Propag..

[9] Ramadan M., Dahle R. (2019). Characterization of 3-D Printed Flexible Heterogeneous Substrate Designs for Wearable Antennas. IEEE Trans. Antennas Propag..

[10] Wu Y., Vallecchi A., Yang Y., You Z., Shamonina E., Stevens C.J., Grant P.S. (2022). 3D Printed Active Origami Dielectrics for Frequency Tunable Antennas Through Mechanical Actuation. IEEE Access.

[11] Tang C., Zheng H., Li Z., Zhang K., Wang M., Fan C., Li E. (2023). Broadband Flexible Microstrip Antenna Array with Conformal Load-Bearing Structure. Micromachines.

[12] Guerron P., Dahle R., Chang Y.H., Noghanian S. (2022). Reconfigurable 3-D Printed HoneyComb Puzzle Piece Patch Antenna. Proceedings of the 2022 IEEE International Symposium on Antennas and Propagation and USNC-URSI Radio Science Meeting, AP-S/URSI 2022—Proceedings.

